# Protein-protein Interaction Networks of *E. coli* and *S. cerevisiae* are similar

**DOI:** 10.1038/srep07187

**Published:** 2014-11-28

**Authors:** S. Wuchty, Peter Uetz

**Affiliations:** 1 Dept. of Computer Science, Univ. of Miami, Coral Gables, FL 33146, USA; 2 Center for Computational Science, Univ. of Miami, Coral Gables, FL 33146, USA; 3 Center for the Study of Biological Complexity, Virginia Commonwealth University, Richmond, VI 23284, USA

## Abstract

Only recently novel high-throughput binary interaction data in *E. coli* became available that allowed us to compare experimentally obtained protein-protein interaction networks of prokaryotes and eukaryotes (i.e. *E. coli* and *S. cerevisiae*). Utilizing binary-Y2H, co-complex and binary literature curated interaction sets in both organisms we found that characteristics of interaction sets that were determined with the same experimental methods were strikingly similar. While essentiality is frequently considered a question of a protein's increasing number of interactions, we found that binary-Y2H interactions failed to show such a trend in both organisms. Furthermore, essential genes are enriched in protein complexes in both organisms. In turn, binary-Y2H interactions hold more bottleneck interactions than co-complex interactions while both binary-Y2H and co-complex interactions are strongly enriched among co-regulated proteins and transcription factors. We discuss if such similarities are a consequence of the underlying methodology or rather reflect truly different biological patterns.

E. *coli* is a primary model organism for microbial biology and applied bacteriology, ranging from studies of fundamental processes to structural genomics and the design of modern antibiotics. Maps of its protein-protein interaction (PPI) network are therefore of utmost importance for our understanding of its basic biological functions. Large-scale high-throughput tandem affinity purification approaches followed by mass spectrometry (AP/MS) have identified the composition of protein complexes in *E. coli*^1^,^2^ but are usually unable to distinguish if two proteins interact either directly or through other intermediaries. In turn, yeast two-hybrid (Y2H) approaches do not reveal the composition of a complex but allow an insight into its binary interactions. Specifically, such a system has been successfully applied to find protein-protein interactions in several eukaryotes^3^,^4^,^5^,^6^,^7^, prokaryotes^8^,^9^, and viruses^10^,^11^. Recently, a first map of binary protein-protein interactions in *E. coli* was released^12^ that has been entirely determined by a yeast two-hybrid approach. To date, however, *S. cerevisiae* remains the best-studied organism whose interactome has been comprehensively investigated by various experimental means^3^,^7^,^13^,^14^,^15^,^16^, allowing a thorough evaluation of the quality of these studies^3^.

Predominantly, we compare the interactome characteristics of different data sets in *E. coli*, accounting for experimentally determined binary-Y2H, co-complex as well as literature curated binary interaction data. Importantly, we observed surprising differences in the underlying data, suggesting that certain characteristics are strongly data set specific. Notably, our *E. coli* specific results strongly resemble analogously made observations in corresponding protein-protein interaction data sets in *S. cerevisiae*.

## Methods

### Protein-protein Interactions

We collected 2,186 binary-Y2H interactions between 1,264 proteins in *E. coli* that were experimentally determined using a yeast-two-hybrid approach (Y2H) by Rajagopala *et al.*^12^. Furthermore, we utilized a total of 9,399 co-complex interactions between 2,044 proteins that were experimentally derived from large-scale tandem affinity purification approaches followed by mass spectrometry (AP/MS) as provided by Hu *et al.*^1^ and Butland *et al.*^2^. To find interactions Hu *et al.* used a logistic regression procedure, accounting for the degree of consistency of co-purified protein pairs. Such an approach balanced the tradeoff between “spoke” and “matrix” representation models of interactions within co-purified groups of proteins to decrease the false discovery rate^1^. Finally, we obtained 1,929 literature-curated binary interactions between 1,399 proteins provided by Rajagopala *et al.*^12^ that were largely curated from small –scale studies and thus obtained by a multitude of methods, including yeast-two hybrid approaches. As a source of binary-Y2H interactions in yeast we utilized 2,930 interactions between 2,018 yeast proteins provided by Yu *et al.*^3^. As for co-complex interactions we used 9,420 interactions between 2,935 yeast proteins that were experimentally derived from large-scale tandem affinity purification approaches followed by mass spectrometry from Krogan *et al.*^13^ and Gavin *et al.*^14^. Specifically, Krogan *et al.* used a machine-learning procedure while Gavin *et al.* employed a “spoke” model to find interactions. As for a literature curated binary set of yeast interactions we used 3,624 PPIs between 1,873 proteins from the HINT database (Aug. 2013)^17^ that were mostly determined using yeast-two hybrid approaches.

### Essential Genes

We used 712 essential proteins in *E. coli* and 1,110 essential genes in *S. cerevisiae* from DEG 10, an update of the database of essential genes (DEG) that collects data about essential genes from the literature^18^. Note that the *E. coli* specific data combines sets from individual studies, each of which reported fewer than 712 essential genes.

### Enrichment Analysis as a Function of Degree

We grouped proteins according to their number of interactions in an underlying protein-protein interaction network. We represented each group by *N*_≥*k*_ proteins that had at least *k* interactions. In each group we determined the fraction of essential proteins, *f*_≥*k*_. As a null model, we sampled random sets of essential proteins of equal size out of all proteins in the underlying interaction network. Specifically, we defined 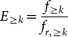 as the enrichment of essential proteins. *f_r,_*_≥*k*_ referred to the corresponding random fraction of essential proteins in the corresponding group where all proteins had at least *k* interactions. After averaging *E* over 1,000 randomizations *E* > *1* pointed to an enrichment and *vice versa*, while *E* ~ *1* indicated a random process^19^.

### Protein Complexes

For *E. coli*, we utilized a set of 517 protein complexes from a co-affinity purification/mass spectrometry (AP/MS) study^1^. As for *S. cerevisiae*, we collected 408 protein complexes from the CYC2008 database (version 2.0, Aug. 2013)^20^.

### Regulatory Interactions

We used 4,442 regulatory interactions between 187 transcription factors and 1,638 genes in *E. coli* from RegulonDB (version 8.0)^21^. As for *S. cerevisiae* we utilized 48,082 regulatory interactions between 183 transcription factors and 6,403 genes from the YEASTRACT database (August 2013)^22^.

### Bottleneck Edges

As a global measure of an edges centrality, we calculated its betweeness centrality, indicating an interactions appearance in shortest paths through the whole network. In particular, we defined betweeness centrality *c_B_* of an edge *e* as 
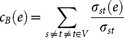
, where *σ_st_* was the number of shortest paths between proteins *s* and *t* while *σ_st_ (e)* was the number of shortest paths running through edge *e*. We defined a set of bottleneck edges as the top 10% of interactions with highest betweeness centrality^23^.

### Interactions between functional classes

Proteins were grouped according to broad functional classes that were defined by clusters of orthologous groups (COGs)^24^,^25^ since COGs provide a consistent classification of bacterial and eukaryotic species based on orthologous groups. Focusing on a set of protein-protein interactions, we counted the occurrence of different class combinations^8^. For each combination of classes *i, j* we determined its probability 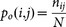, where *N* is the total number of interactions between classes. As a null-model, we determined an expected probability of interactions between classes *i*, *j* 
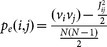
. Specifically, *v_i_* is the number of viable proteins in class *i* (*i.e.* proteins of class *i* that are involved in at least one interaction in the underlying set), and *J_i,j_* is the number of genes that are involved in both classes. Combining these probabilities, we determined a log-odds ratio 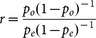. For large samples, we estimated the variance of the odds distribution as 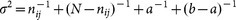 where 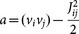 and 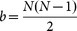. In particular, we calculated a P-value for the significance of a link between two classes by a Z-test, where 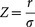. Specifically, we considered each link that had a P < 0.05^8^.

## Results

To compare different sets of interactions in *E. coli*, we collected 2,186 binary interactions between 1,264 proteins that were determined with a yeast-two hybrid approach (binary-Y2H)^12^. Furthermore, we accounted for 9,399 experimentally obtained co-complex interactions^1^,^2^ that connect 2,044 proteins (co-complex). As for literature curated binary interactions, we investigated a set of 1,929 interactions^12^ between 1,399 proteins that were mostly obtained with yeast-two hybrid approaches.

### Essentiality

**Figure 1:**
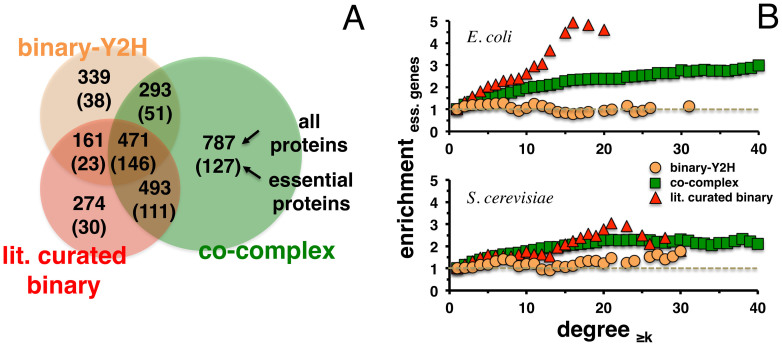
Enrichment of essential genes in different protein-protein interaction datasets of *E. coli.* (A) Overlaps between sets of proteins that are involved in binary-Y2H, co-complex and literature curated binary protein-protein interaction data sets in *E. coli*, including the number of essential proteins that are involved in interactions (brackets). (B) Enrichment of essential bacterial proteins as a function of their number of interactions in *E. coli* and *S. cerevisiae*. Notably, we observed that binary-Y2H interactions failed to show an ascending trend compared to literature curated binary and co-complex interactions in both organisms.

The importance of a protein in a protein-protein interaction network is frequently considered a function of its number of interaction partners. For instance, the so-called centrality-lethality rule^3^,^26^ suggests that central proteins with many interactions are more likely to be essential than poorly connected proteins. While highly connected proteins are more often essential in *S. cerevisiae* they are also involved in an increasing number of protein complexes^27^, suggesting that their essentiality is a consequence of their involvement in essential complexes^28^,^29^,^30^. To determine essentiality-specific characteristics we utilized a set of 712 essential proteins in *E. coli* from the DEG database^18^. While the overlaps between the sets of proteins that are involved in our different protein-protein interaction networks of *E. coli* are considerable, we surprisingly found an enrichment when we focused on the corresponding sets of essential interacting genes (Fig. 1A). In Fig. 1B we determined the enrichment of essential proteins in groups of increasingly interacting proteins in different interaction data sets of *E. coli*. To compare with yeast specific data we used a set of 2,930 binary-Y2H interactions^3^, 9,420 co-complex interactions^13^,^14^ and 3,624 literature curated binary interactions^17^. Similarly to yeast^3^ we observed that essential proteins were no more essential than any other proteins in binary-Y2H protein-protein interactions in *E. coli*.

**Figure 2:**
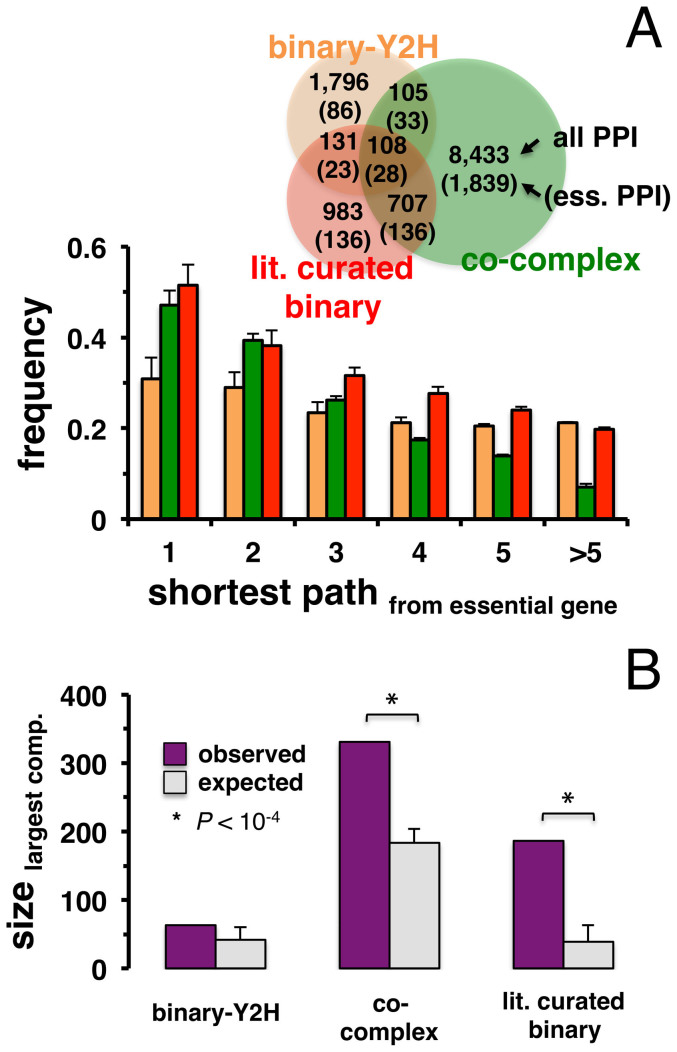
Essential genes in the protein-protein interaction networks of *E. coli*. (A) The Venn diagram shows overlaps between the binary-Y2H, co-complex and literature curated binary interaction networks in *E. coli*, as well as interactions between essential genes in these sets (brackets). (B) We grouped *E. coli* proteins that were placed a given distance away from essential proteins in the underlying interaction networks. The fraction of essential proteins is largest in the immediate vicinity of other essential proteins. Error bars indicate 95% confidence interval. (C) Observed and expected sizes of the largest connected component between essential genes in *E. coli*. As a null model we randomly sampled essential genes 10,000 times, indicating that the size of the largest component in co-complex and literature curated binary interaction sets was significantly larger than randomly expected (P < 10^−4^).

In Fig. 2A, we determined the overlaps of protein-protein interactions in the given data sets, including interactions between essential proteins. While the overlap between interactions in the different sets is limited, interactions between essential proteins appear to further deplete overlaps. Starting from essential proteins, we determined groups of proteins that are a given number of interactions away in the underlying network. In each bin we calculated the fraction of essential proteins, indicating that essential proteins generally accumulated in the immediate vicinity of other essential proteins in all interaction sets of *E. coli* (Fig. 2B). Notably, enrichments of essential proteins in the network vicinity of each other was strongest in co-complex, followed by literature-curated binary and binary-Y2H interactions, an observation that matches results obtained with corresponding interaction data sets in *S. cerevisiae*^3^.

The observation that essential proteins predominantly appear in the vicinity of each other suggested that essential proteins were organized in subnetworks through their interactions. By randomly picking sets of essential genes 10,000 times, we determined the observed and expected sizes of the largest connected components between essential genes. Fig. 2C indicates that both co-complex and literature curated binary interactions in *E. coli* showed significantly larger subnetworks composed of essential proteins than were randomly expected (P < 10^−4^), again a result that matches similar observations in yeast^3^.

### Functional cross-talk

**Figure 3:**
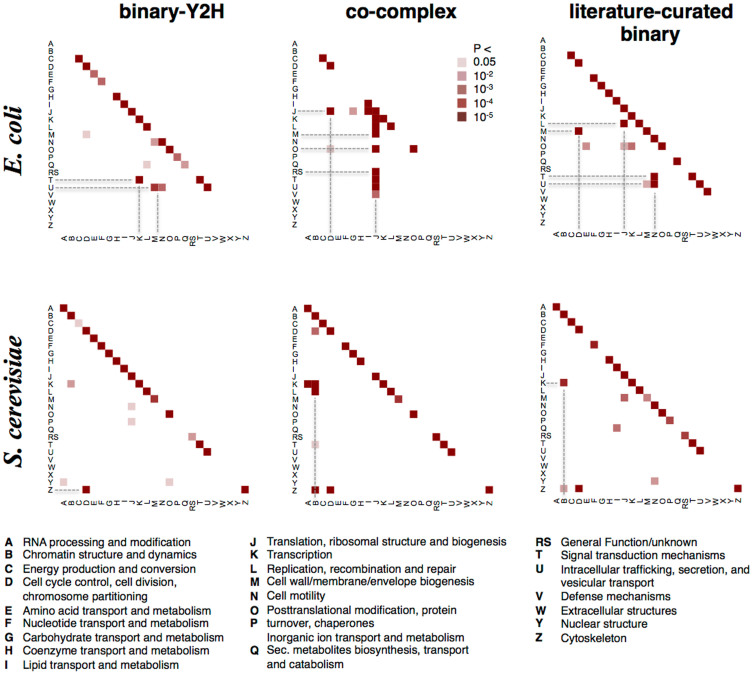
Interactions between functional classes. Significant connections between functional classes are mediated by protein-protein interactions. For each dataset and each class combination a P-value was calculated, reflecting the significance of the interaction density between classes in an interaction dataset of certain size and class coverage. Functional groups that exhibit cross-talk are highlighted (dotted lines are a guide to the eye).

We grouped *E. coli* and yeast proteins according to broad functional classes that were defined by clusters of orthologous groups (COGs)^24^,^25^ and counted the occurrence of inter-class PPIs within the different interaction datasets^8^. We determined a log-odds ratio of the observed and expected frequencies of interactions between proteins of the corresponding functional classes, allowing us to calculate a P-value with a Z-test (see Materials and Methods). Fig. 3 shows that interactions mostly appeared between the same classes in binary-Y2H, co-complex and literature curated binary data. Interestingly, we found significant cross-talk between different functions that was dependent on the species and method used. For instance, both binary-Y2H and co-complex data show enriched interactions among yeast cell cycle (letter “D”) and cytoskeleton proteins (“Z”). However, co-complex and literature-curated binary interactions in yeast also point to interactions between chromatin proteins and transcription/replication proteins/RNA processing (Fig. 3). By contrast, the difference between binary-Y2H and co-complex data is much more pronounced in *E. coli*. Here, binary-Y2H interaction data sets detect the strongest cross-talk signal between transcription and signal transduction while co-complex data indicates a strong connection between translation/ribosomes and a number of other processes. However, given the unusually strong connection of ribosomal proteins to several other processes we suspect that this observation is the consequence of an artifact of ribosome-associated proteins (see discussion).

### Protein Complexes

**Figure 4:**
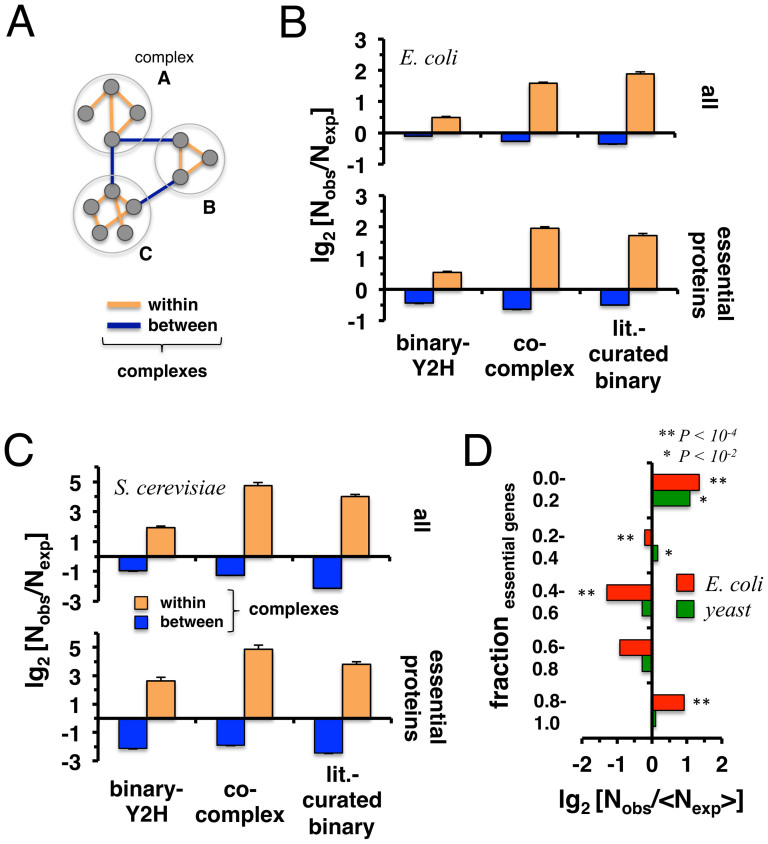
Protein complexes and protein-protein interactions. (A) Schematic illustration of interactions that appear between and within complexes. In (B) *E. coli* and (C) *S. cerevisiae* we determined the number of binary-Y2H and co-complex interactions between proteins in the same complex as well as within complexes. As a random null model we resampled proteins in complexes 10,000 times. Our results in the upper panels indicate that interactions between complexes appear diluted in all interaction sets (P < 10^−4^) while interactions in the same complexes seem to be enriched (P < 10^−4^). Analogously, we investigated interactions between essential proteins in both organisms (lower panels), confirming an enrichment of PPIs within complexes compared to between-complexes in both organisms (P < 10^−4^). In (D) we calculated the fraction of essential genes in each complex. As a null-model, we randomly sampled essential genes 10,000 times, indicating that complexes generally do not randomly contain essential proteins. Notably, complexes with a very low/high fraction appear to show a significant enrichment of essential genes.

As for a different level of cellular organization, we utilized a set of 517 protein complexes in *E. coli* that were obtained from a co-affinity purification study followed by mass spectrometry analyses^1^. As for yeast, we used 408 protein complexes from the CYC2008 database^20^ that collects experimentally obtained complex information from the literature. We wondered whether interactions in our different data sets are enriched within single complexes or between complexes (Fig. 4BC). Considering binary-Y2H and co-complex interactions in both organisms, we counted the number of inter- and intra-complex interactions. As a random null model we randomly assigned the same number of proteins to each corresponding complex 10,000 times. Interactions connecting complexes appeared less frequently than expected in both organisms and data types (P < 10^−4^, Fig. 4BC). In turn, interactions between proteins in the same complex occurred more frequently than expected (P < 10^−4^, Fig. 4BC). Focusing on interactions between essential proteins, we found an even stronger trend in both organisms (P < 10^−4^, Fig. 4BC). In Fig. 4D we calculated the fraction of essential genes in each complex. As a null-model, we randomly sampled essential genes 10,000 times. Notably, complexes that had the lowest and highest fractions of essential genes were significantly enriched in *E. coli*. Such a result was confirmed only for yeast complexes with few essential proteins.

### Regulatory interactions

**Figure 5:**
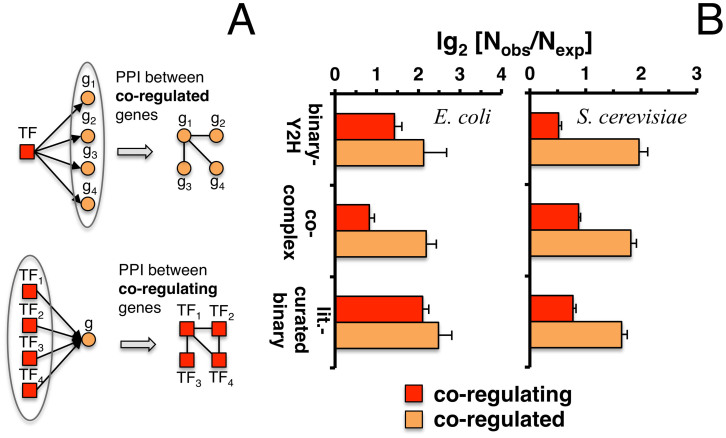
Topological features of protein-protein interaction sets in *E. coli* and *S. cerevisiae*. In (A) we schematically illustrate interactions between co-regulated and co-regulating genes. In particular, co-regulated genes are considered a set of target genes that are controlled by a transcription factor. In turn, co-regulating genes refer to transcription factors that control the expression of the same genes. In (B) we calculated the number of interactions between co-regulated genes. In turn, we also calculated the enrichment of interactions between co-regulating genes in both *E. coli* and *S. cerevisiae*. As for their expected levels, we randomized the set of target genes of each transcription factor 10,000 times. While enrichments were significant in all datasets (P < 10^−4^) transcription factors tend to interact more frequently with each other than their target genes.

Utilizing 4,442 regulatory interactions between 187 transcription factors and 1,638 genes in *E. coli* from RegulonDB^21^, we measured the number of interactions that appeared between co-regulated target genes (Fig. 5A). In addition, we determined the number of interactions between transcription factors co-regulating the same target genes (Fig. 5A). As a null model we randomly assigned the same number of targeted genes to each corresponding transcription factor 10,000 times. Fig. 5B indicates that binary-Y2H as well as co-complex interactions between targets of transcription factors were significantly enriched (P < 10^−4^). Utilizing 48,082 regulatory interactions between 183 transcription factors and 6,403 genes from the YEASTRACT database^22^ we obtained similar results in yeast (P < 10^−4^), confirming that enrichment signals were stronger for interactions between transcription factors than among their target genes^3^.

### Network topology

**Figure 6:**
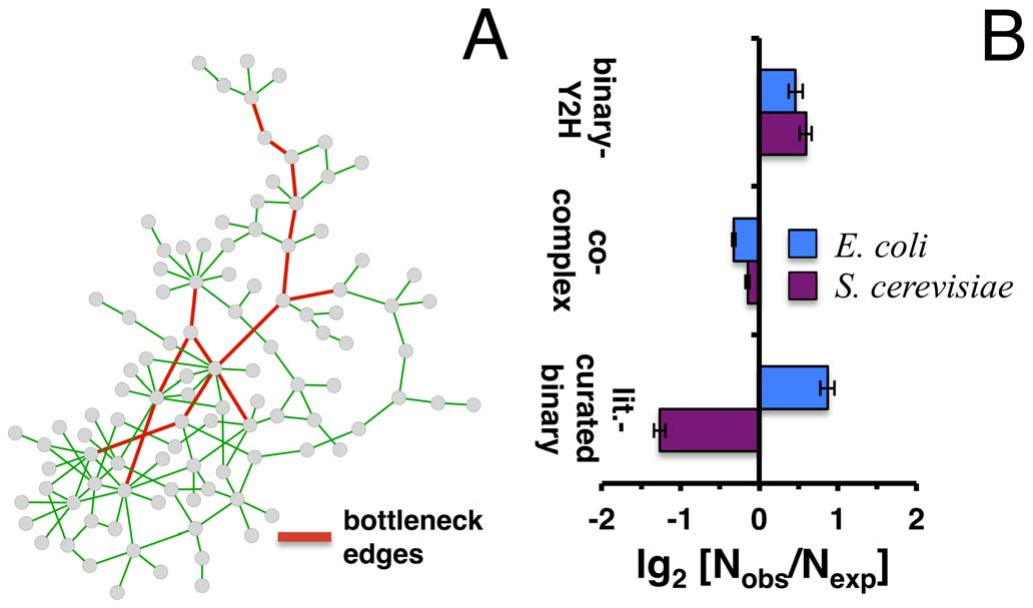
Bottleneck interaction sets in *E. coli* and *S. cerevisiae*. (A) To illustrate the concept of bottleneck edges, we considered a toy network of 138 interactions. After calculating their betweeness centrality we defined the top 10% of edges with highest betweeness as bottleneck set. In (B) we calculated the edge betweeness of proteins in combined networks of binary-Y2H, co-complex and literature curated binary interactions in both *E. coli* and *S. cerevisiae*. Randomizing sets of such bottleneck edges 10,000 times, we calculated the ratio of observed and expected number of bottleneck edges that appeared in the binary-Y2H, co-complex and literature curated binary sets. While enrichments were significant in all cases (P < 10^−4^) we generally observed an enrichment of binary-Y2H interactions, while co-complex interactions appeared to be significantly diluted. However, *E. coli* and yeast differ in literature curated binary PPI data.

As a different measure of the central placement of interactions we calculated their edge betweeness centrality in a network that combined binary-Y2H, co-complex and literature-curated binary interactions in *E. coli* as well as yeast. Specifically, we defined a set of bottleneck interactions^23^ as the top 10% of interactions with highest centrality (Fig. 6A). To assess if bottlenecks were preferably provided by binary-Y2H, co-complex or literature curated binary interactions, we randomly sampled bottleneck edges 10,000 times. Fig. 6B clearly suggests that bottleneck interactions preferably occurred in the binary-Y2H interaction set in both organisms (P < 10^−4^), a result that was previously reported in yeast^3^. Notably, however, we observed that literature curated binary interactions in yeast significantly contributed to bottleneck interactions while we found the opposite in *E. coli*.

## Discussion

Until recently, *S. cerevisiae* remained the only organism whose interactome has been investigated extensively by multiple experimental approaches. However, the availability of novel protein-protein interaction data in *E. coli* from different experimental sources offered a new opportunity to analyze and compare the properties of prokaryotic and eukaryotic interaction networks. In particular, we utilized a recently published set of binary-Y2H interactions that was determined by a yeast two-hybrid method, an interaction set obtained from tandem affinity purification approaches followed by mass spectrometry as well as literature curated binary interactions in *E. coli*. In comparison to yeast specific interactions, we found surprising similarities and differences in the various interaction datasets that were largely congruent on an organism's level.

### Essential proteins

While proteins with many different interaction partners are more often essential we observed that such a trend was absent when we considered binary-Y2H interactions in *E. coli*. Notably, binary-Y2H interactions in yeast are not enriched in essential genes among highly connected proteins either^3^. This observation may be based on the fact that larger complexes are more likely to contain essential components while their size leads to larger degrees, especially when matrix models are used. Both co-complex protein-protein interaction data sets may contain highly connected artifacts because of contaminated purifications or because of unspecific “sticky” proteins, respectively. Specifically, yeast two-hybrid approaches are highly sensitive to expression levels and to auto-activating bait proteins. As a consequence, proteins appear to have many more biologically relevant binary interaction partners than actually exist in reality. Furthermore, yeast two-hybrid approaches may not detect interactions of many essential genes because they may not interact at all with other proteins. For instance, metabolic enzymes may only interact with their small molecule substrates or under certain physiological conditions.

As for properties of essential genes in different interaction data sets, we observed that essential genes largely accumulate in the vicinity of each other, a characteristic that is valid for both organisms. Notably, binary-Y2H interactions fail to produce a statistically significant size of a connected component that was composed of essential genes. In both yeast^3^ and *E. coli* the largest components in binary-Y2H interactions were generally smaller than in co-complex and literature curated binary interactions. The observed difference is clearly a result of the data models used given that the number of interactions increases linearly (spoke) or exponentially (matrix) in AP/MS data. As for literature curated interactions an sociological bias potentially determines degree: the more a protein is considered important the more it is studied, and - as a consequence - more interactions are found.

### Interactions within and between protein complexes

Interactions *within* protein complexes were significantly enriched, while interactions *between* complexes were significantly depleted. When we considered interactions between essential proteins we found that this trend was reinforced in both organisms. Since yeast complexes were reported to show a modular nature of essentiality^30^,^31^,^32^, our results indicate that such a behavior is true for different types of interactions and both pro- and eukaryotes, suggesting that such an observation potentially represents a universal pattern of biological systems.

### Regulatory interactions

Another level of cellular organization is represented by transcription factor – gene interactions. All interaction data sets provided significant enrichments of interactions between targets of the same transcription factor in *E. coli*, a result that strongly resembles observations made in yeast^3^. Notably, interactions between transcription factors that regulated the same target genes were enriched in both organisms, suggesting that transcription factors are preferably wired between each other to carry out regulation of gene expression. Since enrichment signals appear similar in binary and co-complex of both organisms, such transcription factor specific characteristics potentially indicate a universal pattern.

### Network topology

In a combined network of binary-Y2H, co-complex and literature curated binary interactions, we found that binary-Y2H interaction sets in both organisms were significantly enriched for bottleneck interactions while the opposite held for co-complex interactions. Since proteins in co-complex interactions are involved in more interactions, at least theoretically, shortest paths may be more evenly distributed over different edges, therefore providing less bottleneck interactions. Notably, a similar result was previously obtained in yeast^3^ and may be rooted in the experimental way interactions have been detected. As mentioned before, many yeast two-hybrid interactions probably reflect transient interactions between complexes while the matrix model used for complexes over-represents interactions within complexes.

### Conclusions

Only when equivalent datasets are used, we can meaningfully compare protein interaction patterns in prokaryotes and eukaryotes. A new large-scale dataset from *E. coli* allows us for the first time to compare binary interaction data to that of yeast. We find that prokaryotes and eukaryotes (here: yeast) behave surprisingly similar in a number of network characteristics. Y2H and AP/MS studies appear to be more different than datasets differ between prokaryotes and eukaryotes generated by the same methods. We conclude that the *differences* in network characteristics between prokaryotes and eukaryotes are thus likely to be artifacts of the experimental approaches rather than differences in biology.
